# Late stage presentation of HIV-positive patients to antiretroviral outpatient clinic in Zambia

**DOI:** 10.4102/sajhivmed.v18i1.717

**Published:** 2017-11-30

**Authors:** Timothy Martin, Morgan Mweene

**Affiliations:** 1 The School of Medicine, Medical Sciences and Nutrition, University of Aberdeen, United Kingdom; 2 Zambia Medical Association, Lusaka, Zambia; 3 Zambia College of Physicians, Zambia; 4 East Central and Southern Africa College of Physicians, Zambia; 5 International Society of Nephrology, Brussels, Belgium

## Abstract

**Background:**

The World Health Organization (WHO) and the Zambian Ministry of Health set out new guidelines on combination antiretroviral therapy (cART) in 2013 expanding the eligibility criteria for patients with HIV.

**Objectives:**

The primary objective were to determine when cART was initiated in HIV-positive outpatients according to clinical and immunological criteria, and to identify what proportion of patients who were eligible for cART according to 2013 WHO and 2013 Zambian cART guidelines were currently on cART.

**Methodology:**

This was a clinical audit of HIV-positive outpatients attending the cART clinic at Ndola Central Hospital in Ndola, Zambia, with retrospective cross-sectional chart review and survey design. Data were collected from clinical records and interviews with patients.

**Results:**

A total of 99% of patients eligible for cART according to 2013 guidelines were on treatment. Clinical staging of patients at initiated on cART (*n* = 206) was as follows: 28% clinical stage I, 21% clinical stage II, 36% clinical stage III and 15% clinical stage IV. The median CD4 count when patients were started on cART was 147 cells/mm^3^.

**Conclusion:**

The results show that a majority of patients were initiated on cART late in their disease course according to immunological (CD4 < 200 cell/mm^3^) and clinical criteria (stage III or IV). However, the vast majority of patients eligible for cART were currently on treatment. The late initiation of cART appears to be a result of late diagnosis of HIV.

## Introduction

Human immunodeficiency virus (HIV) and acquired immune deficiency syndrome (AIDS) are major causes of morbidity and mortality, with an estimated 36.7 million HIV-infected people worldwide in 2015.^1^ Illness and symptoms associated with HIV are grouped into four clinical stages according to World Health Organization (WHO) criteria, ranging from asymptomatic disease (stage I) to AIDS defining illness (stage IV).^2^ Immunological monitoring of HIV is achieved through a blood test called the CD4 count, which measures the CD4 lymphocytes. There is a strong relationship between CD4 count, immunodeficiency and risk of HIV-related illnesses.^3,4^ Since the late 1980s, various antiretrovirals (ARVs) have been developed which can significantly improve outcomes for people with HIV. ARV drugs are given in combination in triple regimens known as combination antiretroviral therapy (cART).^3^

Zambia is one of the countries most severely affected by the HIV epidemic, with an estimated prevalence of 13.3% in adults in 2013–2014.^5^ The HIV epidemic in Zambia came to the fore in the 1990s; it was a generalised epidemic with the main method of transmission being heterosexual sex.^6^ There are a variety of factors which could explain the HIV epidemic in Zambia. These include a tendency to have multiple concurrent partners, frequent intergenerational sex, low condom usage and transactional sex.^6,7,8^ The prevalence of HIV in Zambia has decreased from 15.6% in 2001–2002 to 13.3% in 2013–2014; however, it remains higher in some regions such as the Copperbelt, where the prevalence was 18.2% in 2013–2014.^5^

Over the past few years, there have been improvements in HIV testing and counselling (HTC) and HIV education programmes in Zambia. There was an increase in HIV testing in public health facilities from 7.4% of adults over 15 years tested in 2008 to 18.1% in 2010, and further increases have occurred since this date under a national HIV policy.^5,6^ Education programmes on HIV in Zambia have helped improve comprehensive knowledge of HIV from 35.9% in 2007 to 42.4% in 2013–2014 in women and from 38.8% in 2007 to 49.0% in 2013–2014 in men.^5^

Access to cART in low-income countries has improved in recent years in large part because of assistance from The Global Fund to Fight AIDS, Tuberculosis and Malaria, and the President’s Emergency Plan for AIDS Relief (PEPFAR).^9^ From 2003, cART was provided free of charge at two centres in Zambia: The University Teaching Hospital in Lusaka and Ndola Central Hospital. This was subsequently expanded to other health facilities throughout Zambia with funding assistance from PEPFAR and the Global Fund, and by 2013, an estimated 82% of people eligible for cART in Zambia received treatment.^6,10^ The national AIDS strategic framework (NASF) set a target of 90% of patients eligible for cART to be on treatment by 2015.^6^

The 2013 WHO ART guidelines recommended that patients with clinical stage III or IV, or a CD4 count of less than 500 cells/mm^3^ should be started on cART.^4^ In 2013, the Zambian Ministry of Health produced a new set of cART guidelines in accordance with the 2013 WHO ART guidelines.^11^ This study was performed in 2014; in 2015, the WHO released new recommendations, which stipulated that cART should be started in all patients with HIV regardless of their clinical stage or CD4 count.^12^

Zambia offers universal free cART treatment to all HIV-positive people who meet eligibility criteria. The Zambian consolidated guidelines for treatment and prevention of HIV infection 2013 guidelines increased eligibility criteria in accordance with the WHO consolidated guidelines on the use of ARV drugs for treating and preventing HIV infection.^4,11^ Earlier initiation of cART has been shown to improve health outcomes for patients with HIV.^13,14,15^ It is important to audit cART use against these new national and international guidelines to ensure their effective use and identify any areas for improvement.

## Methods

### Objectives

The first primary objective of this study was to audit the proportion of patients eligible for cART according to 2013 WHO ART and 2013 Zambian cART guidelines who had been initiated on cART. The second primary objective was to find when HIV-positive outpatients attending the cART clinic at Ndola Central Hospital were initiated on cART according to CD4 count and clinical criteria. CD4 count at initiation of cART will also be stratified against clinical stage. In addition, a subgroup analysis of those started before 01 January 2010 and those started after 01 January 2010 will be performed. The secondary objectives were to (1) establish which cART regimens patients were currently on, (2) ascertain if these regimens were first-line, (3) audit first-line regimens against guidelines, (4) find changes in regimens and reasons for switching, (5) note side effects from the current regimen, (6) find adherence to cART, (7) find prevalence of risk factors for HIV and (8) check reported condom use.

### Study design

A clinical audit of cART regimens with retrospective cross-sectional chart review and survey design.

### Data collection

Information was collected from clinical records and interviews with patients at the adult cART outpatient clinic in Ndola Central Hospital in Ndola, Copperbelt, Zambia. The study population was HIV-positive outpatients attending the adult cART clinic between 11 March 2014 and 07 April 2014. All patients attending the clinic during the study period were invited to participate in the study. The purpose of the study was explained to the patients and written or verbal consent was obtained from patients who wished to participate. In cases where the patient was unable to write their signature but could give verbal informed consent, the data collector noted that verbal consent had been obtained. Patients who did not provide consent were excluded from the study. Information on clinical stage, CD4 count, cART regimens, reasons for changing regimens and side effects from cART was collected from clinical records. Additional information on risk factors for HIV, reported condom use and adherence to medications was collected from interviews with patients. Where patients did not speak English, a translator was used. Information from the data collection sheets was transferred to an Excel spreadsheet for analysis.

### Adherence to guidelines

The 2013 Zambian consolidated guidelines for treatment and prevention of HIV infection and the 2013 WHO consolidated guidelines on the use of ARV drugs for treating and preventing HIV infection both recommend starting cART in any HIV-positive patients with WHO clinical stage III or IV. In addition, these guidelines recommend that patients of any clinical stage with a CD4 count of below 500 cells/mm^3^ should also be initiated on cART.^4,11^ Patients were classified as eligible for treatment if they currently met eligibility criteria according to the 2013 guidelines for cART, or if had previously been started on cART according to guidelines at the time of initiation (Table 1). The eligibility criteria for cART have been expanded; therefore, all patients eligible under previous guidelines would be eligible under the 2013 guidelines with the addition of patients who meet the expanded eligibility criteria. This audit monitored the proportion of patients eligible for cART who were currently on cART to establish the adherence to the 2013 guidelines.

**TABLE 1:** Eligibility criteria for initiation on combination antiretroviral therapy across different time periods.

Time period	ART guidelines applied	Clinical stage	CD4 count (cells/mm^3^)
Before 2010	2006 WHO ART guidelines	Clinical stage III or IV	≤ 200
Between 2010 and 2013	2010 WHO ART guidelines	Clinical stage III or IV	≤ 350
2014	2013 WHO ART guidelines; 2013 Zambian consolidated cART guidelines	Clinical stage III or IV	≤ 500

ART, antiretroviral; WHO, World Health Organization.

ART regimens used in this audit were compared against those recommended as first-line by the 2013 Zambian consolidated cART guidelines and 2013 WHO ART guidelines. The WHO recommended regimens were zidovudine (AZT) + lamivudine (3TC) + nevirapine (NVP); tenofovir disoproxil fumarate (TDF) + 3TC [or emtricitabine (FTC)] + efavirenz (EFV) and TDF + 3TC (or FTC) NVP; with a preference for TDF + 3TC (or FTC) + EFV.^4^ The 2013 Zambian consolidated cART guidelines for first-line regimens were in accordance with WHO guidelines with the addition of abacavir (ABC) + 3TC (or FTC) + EFV regimen.^11^

### Statistics

The sample size required was calculated using the public health statistical programme Epi Info^TM^7. The population size was 9000 as this is the number of HIV-positive patients who attend the cART outpatient clinic at Ndola Central Hospital. The expected frequency of adherence to WHO and Zambian Ministry of Health cART guidelines was set at 90% for initiation of cART. This expected frequency was chosen as 90% was the NASF target for the percentage of patients eligible for cART to be on treatment by 2015.^6^ Using this expected frequency and confidence limits of 5% and a confidence level of 95%, it was calculated that a sample size of 136 was required. Public health statistical programme Epi Info^TM^7 was used for descriptive statistical analyses of this study, including calculating interquartile ranges (IQR) and standard deviations (SD).

## Results

### Epidemiology

A total of 221 patients attended the cART clinic during the study period. Information was collected from a total of 209 patients. The remaining 12 patients did not provide consent to participate, so they were excluded from the study. Of the patients, 60% were females and 40% males (Table 2). The age of the patients ranged from 15 to 70 years; the median age was 43 years (IQR: 36–49); and the mean age was 42 years (SD ± 11). The median age that patients were started on cART was 36 years (IQR: 30–43); for men, this was 40 years (IQR: 34–48) and for women it was 34 years (IQR: 29–40).

**TABLE 2:** Patient demographics.

Patient age groups (years)	Number of men		Number of women		All patients	
	*n*	%	*n*	%	*n*	%
< 25	5	6	16	12.5	21	10
25–34	4	5	17	13.5	21	10
35–44	25	30	49	39	74	35.5
45–54	29	35	35	28	64	30.5
55–64	18	22	7	5.5	25	12
≥ 65	2	2	2	1.5	4	2
All age groups	83	40	126	60	209	-

### Primary outcomes

Of the 209 patients in this audit, 204 met the eligibility criteria through clinical stage or immunological criteria for cART according to the 2013 WHO and Zambian cART guidelines. This was based upon whether patients currently met eligibility criteria or had previously been started on cART according to the guidelines at the time of initiation (Table 1). Four patients had been started on cART previously despite never meeting 2013 cART eligibility criteria. The remaining patient was clinical stage I, but did not have a CD4 count since diagnosis; therefore, it was impossible to determine their cART eligibility. One hundred and two patients met both immunological and clinical criteria, three patients met only clinical criteria and 99 patients were eligible on immunological criteria alone (Table 3).

**TABLE 3:** Patients meeting 2013 combination antiretroviral therapy clinical or immunological eligibility criteria.

2013 ART eligibility criteria met	*n*	%
Number of patients meeting both clinical and immunological criteria (%)	102	49
Number of patients meeting clinical criteria alone (%)	3	1
Number of patients meeting immunological criteria alone (%)	99	47
Number of patients not meeting eligibility criteria (%)	5	2
Total number of patients	209	-

Of the 204 patients who met eligibility criteria, 202 were currently on or had just been started on cART. For the two remaining patients, one was on tuberculosis (TB) treatment and a clinical decision had been made to delay cART initiation; and the other was presenting to the clinic for the first time. The time since initiation of cART ranged from 0 to 200 months, with a median time of 78 months (IQR: 49–96).

There were only two patients (1%) on cART who were eligible for treatment according to the 2013 cART guidelines but would not have been eligible for treatment under the WHO 2010 cART guidelines as their clinical stage was less than III and CD4 count between 350 cells/mm^3^ and 500 cells/mm^3^. There were 27 patients (13%) who were eligible according to 2010 WHO ART guidelines but would not have been eligible under 2006 WHO ART guidelines as their clinical stage was I or II and their CD4 count was between 200 cells/mm^3^ and 350 cell/mm^3^.

**FIGURE 1:**
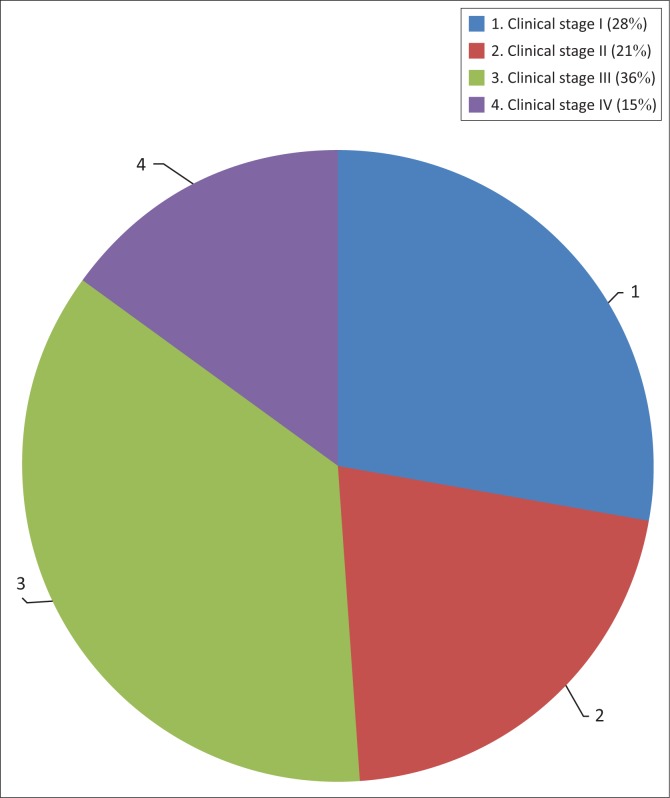
Clinical stage at initiation of combination antiretroviral therapy. (*n* = 206)

The WHO clinical stage of HIV-related illness was available for all 206 patients who were started on cART at the time of initiation (Figure 1). The clinical stages of patients initiated on cART were as follows: 28% (58/206) clinical stage I; 21% (43/206) clinical stage II; 36% (74/206) clinical stage III and 15% (31/206) clinical stage IV. The majority of patients starting cART treatment were clinical stage III or IV, meaning that they were eligible for treatment through the clinical criteria alone.

**FIGURE 2:**
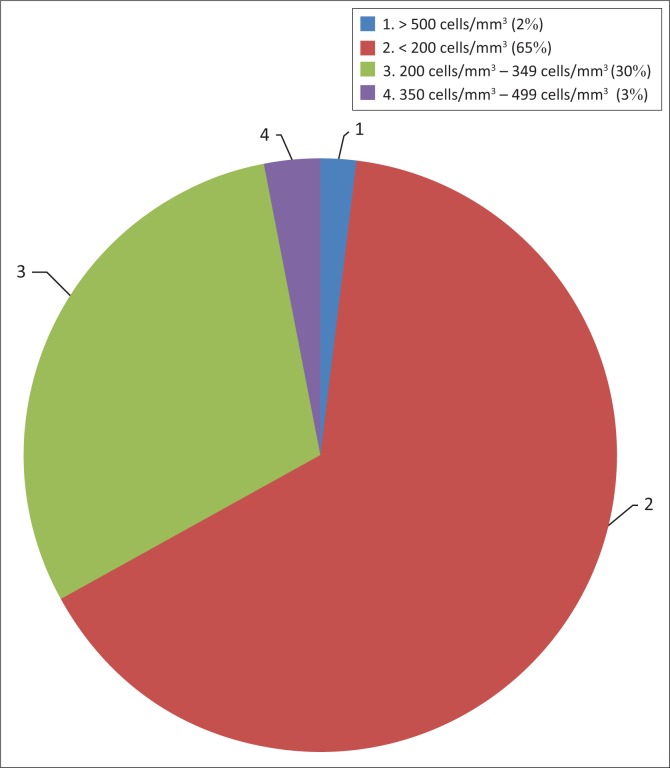
CD4 count of patients at initiation of combination antiretroviral therapy. (*n* = 206)

CD4 count results at initiation of cART were available for 181 of 206 patients and the range was from 1 cells/mm^3^ to 626 cells/mm^3^; the median was 147 cells/mm^3^ (IQR: 84–242) and the mean was 168 cells/mm^3^ (SD ± 117) (Figure 2). Ninety five per cent (172/181) of the patients had a CD4 count of below 350 cells/mm^3^ at initiation of cART, the 2010 WHO criteria for starting cART. Ninety eight per cent (178/181) of patients had a CD4 of less than 500 cells/mm^3^; this is the level set by the 2013 WHO ART guidelines and the 2013 Zambian cART guidelines. The majority of patients were started on cART soon after diagnosis as they were eligible for treatment at diagnosis, 73% of patients were started on cART within one month of diagnosis.

CD4 count at initiation was stratified against clinical stage at initiation of cART to determine if clinical staging was consistent with the degree of immunosuppression; the results are shown in Table 4. No statistical analyses were performed for direct comparison of CD4 count between the groups. However, the median and mean CD4 counts of patients of all clinical stages at initiation on cART do not vary greatly and there is no consistent pattern of increasing CD4 count with increasing clinical stage.

**TABLE 4:** CD4 count (cells/mm^3^) at initiation of combination antiretroviral therapy stratified against clinical stage.

Descriptive statistics	Clinical stage I	Clinical stage II	Clinical stage III	Clinical stage IV
Mean (SD)	192 (±102)	137 (±91)	165 (±133)	170 (±128)
Median (IQR)	185 (103–262)	124.5 (69.5–198.5)	137 (83.5–222)	145 (80.75–246)
Range	47–600	10–410	4–626	1–464

IQR, interquartile ranges; SD, standard deviations.

**FIGURE 3:**
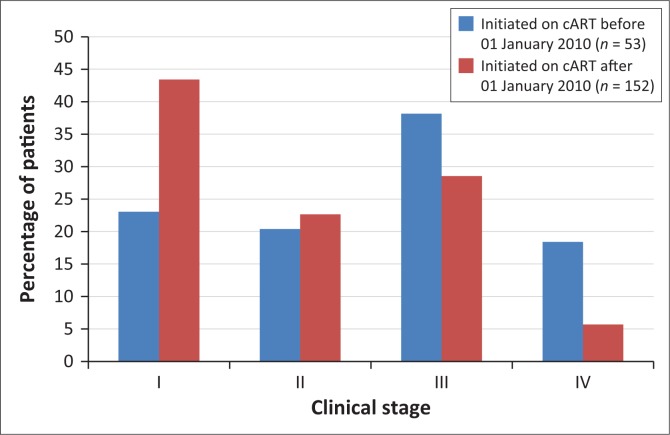
Clinical stage at initiation of combination antiretroviral therapy of patients started before 01 January 2010 and those started after 01 January 2010. (*n* = 206)

As patients in this study had been on cART for varying lengths of time, a subgroup analysis was carried out for two subgroups comprising patients initiated on cART before 2010 and those started after 01 January 2010. The WHO 2006 guidelines applied for patients initiated on cART before 2010 and the WHO 2010 guidelines for patients started on cART between 2010 and 2013 (Table 1). There were seven patients started on cART since 2013 for which the Zambian 2013 cART guidelines would apply; because of the small number, they were put in the subgroup started after 01 January 2010 rather than being given a separate subgroup. One hundred and fifty-three patients had been started on cART before 2010 and 53 since 01 January 2010; there was information on clinical stage at initiation on cART for 152 patients started before 2010 and all 53 patients in the second group (Figure 3). This was a descriptive study and no statistical analyses were performed for comparison between the two groups.

**FIGURE 4:**
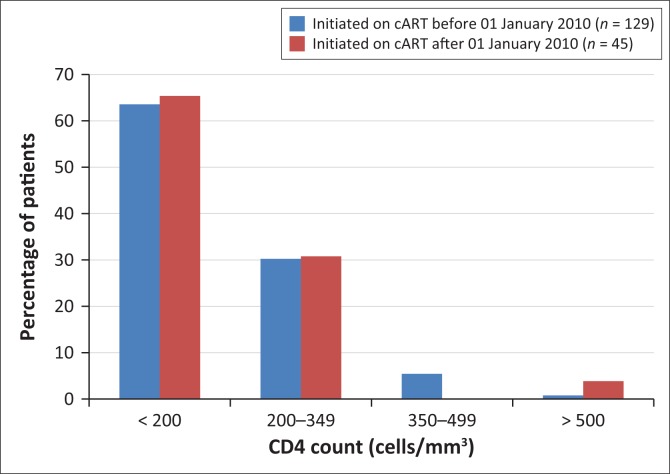
CD4 count at initiation of combination antiretroviral therapy of patients started before 01 January 2010 and those started after 01 January 2010.

CD4 counts at initiation of cART were available for 129 of the 153 patients started before 2010, and for 52 of the 53 patients started since 01 January 2010; the results are shown in Figure 4. For the subgroup started on cART before 2010, the median CD4 count was 152 cells/mm^3^ (IQR: 91–231); the range was from 3 cells/mm^3^ to 572 cells/mm^3^ and the mean CD4 count was 167 cells/mm^3^ (SD ± 110). The subgroup started on cART since 01 January 2010 had a median CD4 count of 145.5 cells/mm^3^ (IQR: 68.25–254.5), a range from 1 cells/mm^3^ to 626 cells/mm^3^ and a mean of 169 cells/mm^3^ (SD ± 134).

### Secondary outcomes

The majority of patients (87%; 180/206) were currently on first-line treatment regimens. Eighty three per cent (149/180) of first-line regimens were those recommended by the 2013 Zambian ART guidelines and 81% (145/180) by WHO 2013 guidelines (Table 5). Sixty three per cent (114/180) of patients on first-line regimens were on the TDF + FTC + EFV regimen which is recommended in preference to other first-line regimens by both the 2013 WHO guidelines and the 2013 Zambian cART guidelines.

**TABLE 5:** First-line combination antiretroviral therapy regimens currently being used.

First-line regimens	Number of patients on each first-line regimen
TDF + FTC + EFV	114
TDF + FTC + NVP	31
ABC + 3TC + EFV	4
First-line regimens not in accordance with guidelines	31

ABC, abacavir; EFV, efavirenz; FTC, emtricitobine; NVP, nevirapine; TC, lamivudine; TDF, tenofovir disoproxil fumarate.

Fifty nine per cent of patients had their cART regimens changed at least once. Fifty per cent of regimen changes were because of side effects of which peripheral neuropathy and lipodystrophy were the most common. The second most frequent reason for regimen change was treatment failure (22%), with immunological treatment failure making up 72% of this total. The third largest group (21%) had their treatment changed to keep regimens in accordance with the Zambian cART guidelines.

Twenty nine per cent of the patients reported having one or more side effects from their current cART regimens. The most common side effects were peripheral neuropathy (in 6% of patients) followed by nausea and/or vomiting (5%) and then dizziness (4%) Peripheral neuropathy was most common in patients on cART regimens containing stavudine.

Of the patients, 202 reported their adherence to cART regimens; 78% reported perfect adherence, indicating that they had not missed a single dose in the last month; 13% reported good adherence, meaning that they had missed one dose in the last month; 6% reported average adherence, meaning that they had missed one dose in the last week; and 2% reported poor adherence, meaning that they had missed more than one dose in the last week (Table 6).

**TABLE 6:** Reported adherence to combination antiretroviral therapy regimens.

Reported adherence	Perfect		Good		Average		Poor	
	%	*n*	%	*n*	%	*n*	%	*n*
Percentage of patients	78	158/202	13	26/202	6	13/202	2	5/202

The most prevalent risk factor amongst the study population was multiple concurrent sexual partners, which was reported by 28% of participants; other risk factors were first sexual contact < 15 years (13%) and transactional sex (11%) (Table 7). Forty eight per cent of participants said they always or usually used condoms, while 48% reported they occasionally or never used condoms, with the remaining patients reporting abstinence over condom use (Table 8).

**TABLE 7:** Risk factors for Human Immunodeficiency Virus.

Risk factors for HIV	Number of patients	
	*n*	%
MSM	4/209	2
IVDU	2/209	1
< 15 at first sexual contact	27/209	13
Transactional sex	22/209	11
Multiple concurrent partners	59/209	28

MSM, Men who have sex with men; IVDU, Intravenous drug use

**TABLE 8:** Reported condom use.

Reported condom use	Always		Usually		Occasionally		Never		Not applicable	
	*n*	%	*n*	%	*n*	%	*n*	%	*n*	%
Number of patients	69/209	33	32/209	15	55/209	26	45/209	22	8/209	4

## Discussion

Ninety eight per cent of patients were eligible for cART using current guidelines, and of these, 99% were currently on cART. These results show that the cART clinic was adhering closely to the 2013 WHO and Zambian national guidelines on initiation of cART.

At initiation on cART, the majority of patients were stage III or IV (Figure 1) and thus eligible for treatment on clinical stage alone.^4,11^ The CD4 counts when patients were started on cART were generally very low (Figure 2), the median being 147 cells/mm^3^, well below 500 cells/mm^3^ the level of eligibility for cART under the 2013 guidelines. These results show that most patients had advanced disease before they were started on treatment, which is likely to have a negative impact on health outcomes.^13,14,15^

CD4 count was stratified against clinical stage to assess if clinical stage was consistent with the degree of immunosuppression. It would be expected that the degree of immunosuppression (measured by decreasing CD4 count) would increase as the clinical stage increased; however, there did not appear to be a clear correlation between the clinical stage and CD4 count in this study (Table 4). This could be because patients all clinical stages had severe immunosuppression (CD4 < 200 cells/mm^3^), meaning that even those who were asymptomatic would also be at high risk of developing diseases or symptoms of clinical stage III or IV. It could also suggest that there were inaccuracies in how a patient’s clinical stage was assessed.

Patients attending the clinic had been on cART for varying time periods; therefore, a subgroup analysis was performed. No statistical tests were performed to compare the subgroups; so it is not possible to make meaningful comparisons between the two subgroups. However, it appeared that a lower proportion of patients started on cART after 01 January 2010 were clinical stage III or IV than those started before 2010 (Figure 3); this could suggest that patients were being diagnosed earlier. Further research is needed with statistical power to test if there has been progress towards earlier initiation of cART.

Since this audit was conducted, the WHO released additional guidelines in 2015 which stipulate that cART should be started in all patients with HIV irrespective of their clinical stage or CD4 count. This would have affected the cART eligibility for five patients in this audit; however, four of these patients had already been started on cART despite not meeting eligibility criteria; the reasons for these patients being initiated on cART were not clear. It appears that late presentation to the cART clinic is the main factor delaying initiation of cART, as once patients attended the cART clinic their eligibility was assessed according to current guidelines and those that were eligible started on cART within a short period, the majority within a month of diagnosis.

Late initiation on cART may occur because of delay in referral to cART services from HIV counselling and testing (HCT) facilities, or late diagnosis as a result of low uptake of HCT programmes. This may mean that patients are only tested for HIV once they become symptomatic by which stage the patient’s illness has already progressed significantly. Recent data have shown an increase in HIV testing in public health facilities. Further research is needed to investigate if these improvements in HCT result in earlier initiation on cART. It is important that these improvements in HIV testing are monitored to ensure they are maintained, and further progress made. In addition, good education about HIV is needed to improve uptake of HCT by informing people of the importance of early diagnosis and assisting early identification of symptoms associated with HIV. HCT facilities also need to have good referral systems to cART services for HIV-positive people as this can be another source of delay in initiation of cART. Early initiation of treatment can improve morbidity and mortality in patients with HIV and additionally reduce the infectivity, minimising further transmission.^13,14,15,16^

A large majority (87%) of patients on cART were currently on first-line regimens of which 83% were in accordance with the Zambian Ministry of Health guidelines (Table 3). There seems to be a concerted effort to change patients onto regimens recommended by the national guidelines, with patients recently started on cART being initiated on the TDF + FTC + EFV regimen and patients on older regimens being changed to regimens in accordance with the Zambian cART guidelines; this is a common reason for regimen change. Just under one third of patients experienced at least one side effect on their current cART regimen, the most common being peripheral neuropathy, which was most common in those on stavudine regimens. The 2013 guidelines no longer recommend regimens with stavudine; this should help reduce the incidence of peripheral neuropathy as a side effect of ARVs.

The most common reported risk factor for HIV in this study was having multiple concurrent partners with 28% of patients reporting this (Table 5). In the Zambian demographics and health survey (ZDHS) from 2013 to 2014, 1.0% of women and 12.8% of men aged 15–49 reported having concurrent sexual partners within the last 12 months.^17^ The prevalence of concurrent sexual partners appears higher in patients attending the ART clinic than in the general population in Zambia. In this study, 11% of patients reported transactional sex; in the ZDHS from 2013 to 2014, 12.8% of men reported paying for sex; however, there is limited information on the number of sex workers in Zambia.^17^ Thirteen per cent of patients reported early sexual contact, the ZDHS from 2013 to 2014 found 11.7% of women and 16.2% of men aged 15–24 years reported sexual contact below the age of 15 years; this is similar to the prevalence in our study. Two per cent of patients in this study reported men who have sex with men (MSM) and 1% intravenous drug use (IVDU); there is currently very limited information on these key populations. However, the population council and partners are currently conducting research in this area of which the results are awaited.^18^ In addition, almost half of the patients in the study reported that they only used condoms infrequently (Table 6). The ZDHS in 2013–2014 reported that 23.1% of HIV-positive people aged 15 years – 49 years used a condom in their last sexual intercourse and 12.1% reported not using a condom.^17^ Low condom usage is likely to result in higher transmission rates in Zambia. It is likely improved education programmes including those targeted at groups of high risk for HIV would be valuable in limiting the further spread of HIV. The most recent Zambia country report on HIV reported that comprehensive knowledge of HIV had increased.^5^ However, further improvements regarding HIV knowledge are crucial and education interventions targeting people with HIV could be particularly effective in reducing transmission.

Other studies have looked at stage of initiation on cART in sub-Saharan Africa and had similar findings. In a study by Mhozya et al. in Tanzania with 366 patients which took place in 2013, 63% of patients presented to the ART clinic with clinical stage III or IV, and 42% with a CD4 count below 200 cells/mm^3^.^19^ Kigozi et al. conducted a study in Uganda in 2007 with 2584 patients where 40% of patients were stage III or IV when started on cART; both these studies showed late initiation of cART; further suggesting this is a significant problem.^20^

There were a number of limitations to this study. No sampling interval or random sampling was used; instead all patients attending the cART clinic during the study period were invited to participate. It would have been preferable to use a systematic or random sampling technique over a longer period to obtain the same sample size. This error in sampling technique may have resulted in an unrepresentative sample, and thus biased the results. From the population attending the cART outpatient clinic (9000) for Ndola Central Hospital, if all patients were seen once every year, there would be an expected attendance of around 1000 patients over the six week period of the study; however, there were only 221 patients who attended the cART clinic during the study period. It is not clear why the number of patients attending the clinic during the study period was so low compared with the expected number; this could be because of non-attendees at the clinic, or the time of year of the study (the end of rainy season) which could mean patients are prevented from attending the clinic. This may make the sample less representative of the population who attend the cART clinic, particularly as there could be bias towards patients who regularly attend the cART clinic. Some interviewees had problems understanding the questions because of language difficulties; some of the interviews were conducted with the help of translators; it is possible that these factors could lead to inaccurate answers. In some cases, information was missing from the notes, and where it was given, dates were sometimes omitted; this made it difficult to obtain a full data set for every patient, thus reducing reliability of results. There were no statistical analyses of reported risk factors which reduce the significance which can be attached to these results. Results on self-reported adherence to cART did not use a prior validated measure of adherence; this may reduce the reliability and comparability of these results. In addition, self-reported adherence often overestimates medication adherence.^21^

## Conclusion

Early initiation of cART can stop disease progression and improve long-term health outcomes. In 2013, new WHO and Zambian national guidelines on cART expanded the eligibility criteria for cART treatment. This study evaluated and audited the use of cART at the outpatient cART clinic at Ndola Central Hospital in Ndola, Zambia, comparing and analysing results against the 2013 Zambian cART guidelines and the 2013 WHO ART guidelines.

The study showed that almost all patients eligible for cART according to the 2013 cART guidelines were on treatment, the majority on appropriate cART regimens; however, many patients were initiated on cART late according to clinical and immunological criteria. This late diagnosis may be because of a lack of uptake of HCT; however, there have been improvements in HCT in Zambia over the past few years. Further research is also needed to assess the impact of increased HCT on earlier initiation of cART. It is important that HIV testing is monitored closely to ensure that testing levels are maintained and further increased, as well as good referral systems to cART clinics.

In conclusion, almost all eligible patients who attended the clinic were started on cART and in the majority of cases cART treatment was initiated soon after diagnosis. Initiation of cART tends to be late according to immunological and clinical criteria, which is probably because of late diagnosis of HIV. Interviews of patients highlighted the limited awareness of the influence of sexual practices and the need to take precautions to avoid the spread of HIV. Progress in tackling HIV requires continued expansion of HCT coupled with education programmes targeting groups at high risk.

## Ethical consideration

A research proposal was approved by the hospital ethics committee. Each patient was assessed to check that they understood what participation in the study would involve. Written or verbal consent was obtained before participation in the study as explained above. Patients were informed that data that would be collected both from interviews and from their clinical records. All information was kept confidential and anonymous.
